# Biochar Alleviates Phytotoxicity by Minimizing Bioavailability and Oxidative Stress in Foxtail Millet (*Setaria italica* L.) Cultivated in Cd- and Zn-Contaminated Soil

**DOI:** 10.3389/fpls.2022.782963

**Published:** 2022-03-25

**Authors:** Xirui Kang, Na Geng, Xu Li, Jinpeng Yu, Hui Wang, Hong Pan, Quangang Yang, Yuping Zhuge, Yanhong Lou

**Affiliations:** National Engineering Research Center for Efficient Utilization of Soil and Fertilizer Resources, College of Resources and Environment, Shandong Agricultural University, Tai’an, China

**Keywords:** heavy metal pollution, biochar, pyrolysis temperature, foxtail millet, Cd immobilization

## Abstract

Soil contamination with multiple heavy metals is a global environmental issue that poses a serious threat to public health and ecological safety. Biochar passivation is an efficient and economical technology to prevent heavy metal contamination of Cd; however, its effects on compound-contaminated and weakly alkaline soil remain unclear. Further, the mechanisms mediating the immobilization effects of biochar have not been evaluated. In this study, three biochar treated at different pyrolytic temperatures [300°C (BC300), 400°C (BC400), and 500°C (BC500)] were applied to Cd-/Zn-contaminated soils, and their effects on plant growth, photosynthetic characteristics, Cd/Zn accumulation and distribution in foxtail millet were evaluated. Further, the effect of biochar application on the soil physicochemical characteristics, as well as the diversity and composition of the soil microbiota were investigated. Biochar significantly alleviated the phytotoxicity of Cd and Zn. DTPA (diethylenetriamine pentaacetic acid)-Cd and DTPA-Zn content was significantly reduced following biochar treatment *via* the transformation of exchangeable components to stable forms. BC500 had a lower DTPA-Cd content than BC300 and BC400 by 42.87% and 39.29%, respectively. The BC500 passivation ratio of Cd was significantly higher than that of Zn. Biochar application also promoted the growth of foxtail millet, alleviated oxidative stress, and reduced heavy metal bioaccumulation in shoots, and transport of Cd from the roots to the shoots in the foxtail millet. The plant height, stem diameter, biomass, and photosynthetic rates of the foxtail millet were the highest in BC500, whereas the Cd and Zn content in each organ and malondialdehyde and hydrogen peroxide content in the leaves were the lowest. Moreover, biochar application significantly increased the abundance of soil bacteria and fungi, as well as increasing the fungal species richness compared to no-biochar treatment. Overall, biochar was an effective agent for the remediation of heavy metal-contaminated soil. The passivation effect of biochar exerted on heavy metals in soil was affected by the biochar pyrolysis temperature, with BC500 showing the best passivation effect.

## Introduction

The presence of heavy metals has caused the deterioration of soil structure, marked decrease in vegetation coverage, and dramatic decline in microbial biodiversity (Lei et al., 2016), besides posing a substantial potential risk to food safety (Zhang et al., 2015). According to the First National Soil Pollution Survey of China for the period of 2005–2013, heavy metal pollution, occurring due to rapid industrialization, urbanization, and intensive agriculture, accounted for 82.8% of the total contamination (Cui et al., 2018). Of the various heavy metal contaminants, Cd is one of the most toxic pollutants and has been categorized as a Class-I carcinogen (Pramanik et al., 2021). Notably, combined Cd/Zn pollution occurs frequently. Zn is an essential trace element for plants that plays essential roles in regulating cell division and proliferation; however, excessive Zn levels can be toxic to plants and animals (Zhang et al., 2020). Furthermore, the uptake and absorption of heavy metals by living organisms occurs with ease, resulting in subsequent poisoning and the development of various diseases (Vareda et al., 2016).

Passivation is a simple, cost-effective, eco-friendly, and sustainable approach and is widely used for heavy-metal remediation (Lu et al., 2017; Zhao et al., 2021). This technique is particularly suitable in regions with a high population and an extremely prominent divide between the shortage of high-quality cultivated land resources and the demand for grain production. This technique could be used for the cultivation of non-grain crops, or in regions in which the phytoremediation of heavy metal-contaminated farmland is not a feasible strategy. Owing to its liming effects, high cation-exchange capacity (CEC), porous structure, and the presence of abundant mineral elements, biochar is a promising material for the remediation of heavy metal-polluted soil (Roy et al., 2021a). Biochar has been shown to exert passivation effects on Cd (Gu et al., 2020), Pb (Boostani et al., 2021), Zn (Sikdar et al., 2020), and Cu (Silva et al., 2019). Biochar application also ameliorates heavy metal-induced damage and enhances the growth of rice (Irshad et al., 2020), wheat (Ali et al., 2019), moso bamboo (Wang et al., 2019), and tobacco (Ren et al., 2021). Jegan et al. (2020) indicated that biochar can decrease the water-soluble Zn and Cd in soil to decrease their cytotoxicity. Biochar derived from sugar cane straw was capable of reducing the availability of Zn, Cd, and Pb in mine-polluted soils and reduce heavy metal uptake in jack bean plants (Das et al., 2020). Furthermore, biochar produced at elevated temperatures of 700°C more effectively decreased the bioavailability and leachability of heavy metals than the biochar produced at low temperatures of 300°C (Ahmad et al., 2016). Similarly, Song et al. (2014) revealed that adding sewage/sludge-derived biochar, produced at 450°C, resulted in more higher plant uptake of Zn than adding biochar produced at 500°C and 550°C. Hence, the effectiveness of biochar in heavy metal immobilization depends on biochar properties, the metal species intended to be immobilized, pyrolytic temperature, as well as the soil and plant type (Rehman et al., 2016; Wu et al., 2017a). However, most studies have focused more on acidic soil and less on the alkaline soil. Therefore, the passivation effect of biochar in alkaline soil warrants further investigation.

Foxtail millet (*Setaria italica* L. Beauv) is an important and popular cereal crop that originates from China and is widely cultivated in India, Nigeria, Japan, and Australia (Sun et al., 2021). It is considered as an excellent strategic reserve crop and a source of food security because of its abundance in nutrients, such as proteins, vitamins, and fatty acids, as well as its drought-tolerance, salinity-tolerance, resistance to fungal infections, and stable grain yields (Han et al., 2019). However, comparatively, its heavy metal tolerance remains unclear (Han et al., 2018).

In this study, we investigated changes in heavy metal (Cd and Zn) speciation and bioavailability in Cd-/Zn-contaminated soils treated with three types of biochar, which were produced at different pyrolytic temperatures, and assessed the physiological effects and changes in gene expression in foxtail millet. Additionally, we evaluated the effects of biochar application on soil enzymes and the microbial community. Our findings may provide insights into the selection of the most appropriate pyrolytic temperatures for biochar processing for heavy metal immobilization, enhancing the passivation-based remediation mechanism of biochar in weakly alkaline soil.

## Materials and Methods

### Soil and Biochar

Experimental soil was collected from the top layer (0–20 cm) of farmland contaminated with Cd and Zn in Shandong Province (China). Soil pH was 8.05; organic matter content was 18.18 g kg^−1^; total N content was 1.52 g kg^−1^; available P and K content was 15.53 and 203.85 mg kg^−1^, respectively; and total Cd, total Zn, DTPA-Cd, and DTPA-Zn content was 0.83, 407.00, 0.18, and 90.32 mg kg^−1^, respectively.

Corn straw biochar were pyrolyzed at 300°C, 400°C, and 500°C (Zhilianrong Technology Co., Ltd., Nanjing, China). The biochar were crushed and passed through a 0.25 mm plastic sieve. The total elemental composition (C, H, and N) was evaluated using a CHN analyzer (Flash-EA112; Thermo Finnigan). Imaging analysis was conducted using scanning electron microscopy (SEM; FEI QUANTA FEG 650). The surface functional groups of the biochar were analyzed using Fourier-transform infrared (FTIR) spectroscopy (Bruker Alpha-Eco ATR-FTIR; Bruker Optics Inc.). Pressed pellets of a mixture of biochar and potassium bromide (1:100, w/w) were scanned 25 times to obtain FTIR spectra with a wavelength resolution of 4 cm^−1^, varying between 400 and 4,000 cm^−1^.

### Plant Cultivation and Experimental Design

Seeds of foxtail millet (Jigu 19, a widely and largely cultivated variety, with excellent Cd tolerance illustrated by our previous research) were obtained from the Institute of Crop Sciences, Shandong Academy of Agricultural Sciences, China. Foxtail millet seeds (2 g) were sown in each plastic pot (diameter, 20 cm; height, 20 cm), filled with 5.0 kg soil containing various types of biochar at 2% application level. Four treatments were conducted as follows: CK, no biochar addition (control); BC300, addition of 300°C-processed biochar; BC400, addition of 400°C-processed biochar; and BC500, addition of 500°C-processed biochar. Three replicates were performed for each treatment, and the different biochar were randomly distributed. The experiment was conducted during the growing season, from June to September 2020. The seedlings were watered every 3 days and cultivated for 115 days.

### Measurement

#### Growth Characteristics and Biomass

Plant height (cm) was measured from the surface of the soil to the natural tip of the plant. Leaf length (cm) was measured from the lamina tip to the point of the petiole intersection along the midrib. The leaf width (cm) was measured from edge to edge at the widest part of the lamina. Leaf length and width of five randomly selected leaves were measured with a ruler at the seedling stage. The stem diameter (mm) was measured at the basal part of the stem using a Vernier caliper at the jointing stage. The soil plant analysis development (SPAD) value was measured using an SPAD-502 chlorophyll meter at the seedling, jointing, and booting stages.

The plants were harvested and washed with tap water to remove all visible fine soil particles. Samples were then rinsed with deionized water, and the roots were dipped in ethylenediaminetetraacetic acid (EDTA) to remove metal ions from the external surface. The roots, stems, leaves, and spikes of the plants were separated and then oven-dried at 70°C until a constant weight was achieved. The dry biomass of the roots, stems, leaves, and spikes was measured using a digital weighing balance (AUW 120 D; Shimadzu Corporation, Japan).

#### Leaf Photosynthesis and Gas Exchange

The second, fully expanded leaves from the tips were used for conducting a leaf gas exchange assay between 09:00 and 11:00 on day 60 after planting, using a portable photosynthetic system (Li-6400XT, LI-Cor, NE, United States). The conditions during the assay were as follows: light intensity of 1,000 μmol m^−2^ s^−1^, leaf temperature of 25°C ± 0.5°C, and a CO_2_ concentration inside the leaf chamber ranging from 300 to 310 mM. Five biological replicates were performed for each treatment. The photosynthetic rate (Pn), stomatal conductance (*G*_s_), intercellular CO_2_ concentration (Ci), and transpiration rate (E) were measured.

#### Enzymatic Activity and Nonenzymatic Antioxidants

Levels of total protein (TP), hydrogen peroxide (H_2_O_2_), anti-superoxide anions, and malondialdehyde (MDA), and the activities of superoxide dismutase (SOD), peroxidase (POD), catalase (CAT), and glutathione reductase (GR) were measured using A045-2, A064-1, A052-1, A003-3, A001-1, A084-3, A007-1, and A062-1 assay kits (Nanjing Jiancheng Bioengineering Institute). The total proline (Pro) content was determined as described by Bates et al. (1973).

#### Total RNA Extraction and Gene Expression Analysis

Total RNA was collected from fresh leaves was performed using TRIzol Reagent (TransGen Biotech, Beijing, China). The quality and quantity of isolated RNA samples were evaluated using 2% agarose gel electrophoresis and spectrophotometry with the DeNovix DS-11. The cDNA was synthesized from 1 μg total RNA using a PrimeScript RT reagent kit with a gDNA Eraser (TakaRa, Tokyo, Japan) according to the manufacturer’s instructions. The transcript levels of genes were assessed using quantitative real-time polymerase chain reaction (qRT-PCR) using SYBR Green Master Mix, and an Applied Biosystems 7,500 Real-time PCR System (Applied Biosystems, CA, United States). The primer sequences are listed in Table 1.

**Table 1:** Primers used for quantitative reverse transcription–PCR.

Gene	Forward primer sequence	Reverse primer sequence
*MnSOD*	TATCATGCAGCTCCACCACCAGAA	TTCCTTGGTGGGAGCCAGATTCTT
*CAT*	TCAAGATCGGTGGAGCGAATCACA	AGGTCTTGGTAACATCAAGCGGGT
*GR*	TGTGTGCTTCGTGGATGTGTT	CCAGTCATGCTTCGGATCAGT

#### Heavy Metal Content in Plants

Dry samples from the roots, stems, leaves, and spikes were ground and passed through a 1 mm sieve. One gram of each subsample was subjected to treatment in a polytetrafluoroethylene crucible using a Marv Furnace at 490°C for 40 min. Subsequently, the material obtained (gray in color) was transferred into a preheated tube, mixed with 10 ml of a concentrated acid solution (HCl:HNO_3_ = 3:1, v/v), and incubated at 100°C for 2 h using a graphite furnace digester (SH230N; Hanon Instruments, Jinan, China) until a clear solution was obtained. The digested solution was then filtered, and the concentrations of Cd and Zn in the digested solution were determined using inductively coupled plasma-mass spectrometry (ICP-MS; Agilent, United States).

#### Soil Heavy Metal Content, Fractionation, and Diethylenetriamine Pentaacetic Acid (DTPA)-Extractable Metal Concentrations

Total Cd and Zn content was digested by treatment with HCl-HNO_3_-HClO_4_ (Chao et al., 2017). Heavy metal fractionation was determined using the BCR three-step sequential extraction procedure described by Li et al. (2020). Available metals were subjected to extraction with DTPA (Abuzaid et al., 2020). The metal content of these extracts was also measured using ICP-MS.

#### Soil Enzyme Activity

Following the methods described by Gu et al. (2019), soil CAT activity (mg KMnO_4_ kg^−1^) was determined by measuring the reduction in H_2_O_2_, using a titration of H_2_O_2_ with 0.1 M KMnO_4_, after the samples were shaken and processed. Briefly, the soil sample (5 g) was mixed with 100 ml of distilled water and subjected to shaking conditions for 30 min. Urease activity (mg NH_4_^+^ kg^−1^ h^−1^) was measured by determining the concentration of NH_4_^+^ released in the hydrolysis reaction after samples were incubated with urea (1%) for 24 h at 38°C. Soil phosphatase activity (mg phenol kg^−1^ h^−1^) was estimated by determining the amount of phenol released after samples were incubated with phenyl disodium phosphate (0.5%) for 24 h at 37°C. Soil sucrase activity was determined using the colorimetric method with 3,5-dinitrosalicylic acid (Yang et al., 2020).

#### DNA Extraction and Sequencing

Bacterial and fungal DNA extraction from 0.2 g soil was conducted using a DNeasy PowerSoil Kit (Qiagen, Inc., Netherlands), and the quality of the extracted DNA was checked using agarose gel electrophoresis. To identify bacterial species in the samples, the V3–V4 hypervariable regions of the 16S rRNA gene were amplified using the primers 338F (5′-ACTCCTACGGGAGGCAGCA-3′) and 806R (5′-GGACTACHVGGGTWTCTAAT-3′). The internal transcribed spacer (ITS) regions of the fungal gene were amplified using the primers ITS5F (5′-GGAAGTAAAAGTCGTAACAAGG-3′) and ITS1R (5′-GCTGCGTTCTTCATCGATGC-3′). Thermal cycling conditions for determining members of the bacterial community comprised initial denaturation at 98°C for 2 min, followed by 25 cycles of denaturation at 98°C for 15 s, annealing at 55°C for 30 s, and extension at 72°C for 30 s, with a final extension at 72°C for 5 min. To identify members of the fungal community, thermal cycling conditions comprised initial denaturation at 98°C for 5 min, followed by 25 cycles of denaturation at 98°C for 30 s, annealing at 52°C for 30 s, and extension at 72°C for 1 min, with a final extension at 72°C for 5 min.

PCR amplicons were purified using Agencourt AMPure Beads (Beckman Coulter, Indianapolis, IN, United States) and were quantified using a PicoGreen dsDNA Assay Kit (Invitrogen, Carlsbad, CA, United States). The amplicons were pooled in equal amounts, and paired-end, 2 × 300 bp sequencing was performed using an Illumina MiSeq platform with MiSeq Reagent Kit V3.

#### Sequence Analysis of 16S and 18S rRNA Amplicons

Raw sequence data were processed and analyzed using QIIME (version 1.8.0) according to the methods described by Pan et al. (2020). Notably, sequences with a length less than 150 bp, with average Phred scores were less than 20, with ambiguous bases, or with mononucleotide repeats of over 8 bp, were eliminated. Then, high-quality sequences were clustered into operational taxonomic units (OTUs) at 97% sequence similarity using UCLUST. OTU classification was conducted using BLAST searches for representative sequences using the Greengenes Database and UNITE database version 6 for the identification of bacterial and fungal species, respectively (Ma et al., 2021).

### Statistical Analysis

All data were processed using Microsoft Excel 2019 and analyzed using the SPSS package (version, 17.0; SPSS Inc., Chicago, IL, United States). Each data point indicated in the figures and tables represents an average value of three replicates. Analysis of variance was performed for the datasets. Mean values of significant differences were separated using *t*-tests or Duncan’s multiple range tests at a significance level of *p* < 0.05.

The Cd and Zn extraction coefficients (ECs), and the bioconcentration factors and translocation factors (BF and TF, respectively) were calculated as follows (Wang et al., 2020):

*EC = total metal in plant/total metal in soil.*

*BF_shoot_ = C_shoot_/C_soil_.*

*BF_root_ = C_root_/C_soil_.*

*TF = C_shoot_/C_root_.*

where *C*_shoot_ and *C*_root_ represent the shoot and root Cd or Zn concentrations (mg kg^−1^), respectively, and *C*_soil_ represents the soil Cd or Zn concentration (mg kg^−1^).

The α-diversity was used to assess the complexity of species diversity for each sample through the Chao1, Shannon, and Simpson indices, which were calculated using QIIME (version 1.8.0). Spearman correlation coefficients between soil environmental factors and microbial community structures were depicted as heatmaps. The sequencing data of bacterial and fungal species were uploaded in the Sequence Read Archive database of the NCBI, with retrieval numbers PRJNA815490 and PRJNA815492, respectively.

## Results

### Characterization of Biochar

The pH, C content, C/H, and C/N increased in the order of BC300 < BC400 < BC500, while the H content decreased in the order of BC300 > BC400 > BC500 (Table 2). A lower N content was observed in BC500 than in BC300 and BC400, and the S content in BC300 was higher than that in BC400 and BC500.

**Table 2:** pH value and elemental composition of the biochars.

Biochar	pH	C %	H %	N %	S %	C/H	C/N
BC300	7.96c	44.59c	3.44a	1.56a	1.1a	12.97	28.59
BC400	9.64b	49.34b	2.50b	1.63a	0.32b	19.70	30.21
BC500	10.30a	59.13a	1.20c	1.41b	0.40b	49.44	41.84

*Lowercase letters in the same column of each treatment are significantly different at p < 0.05*.

**Figure 1:**
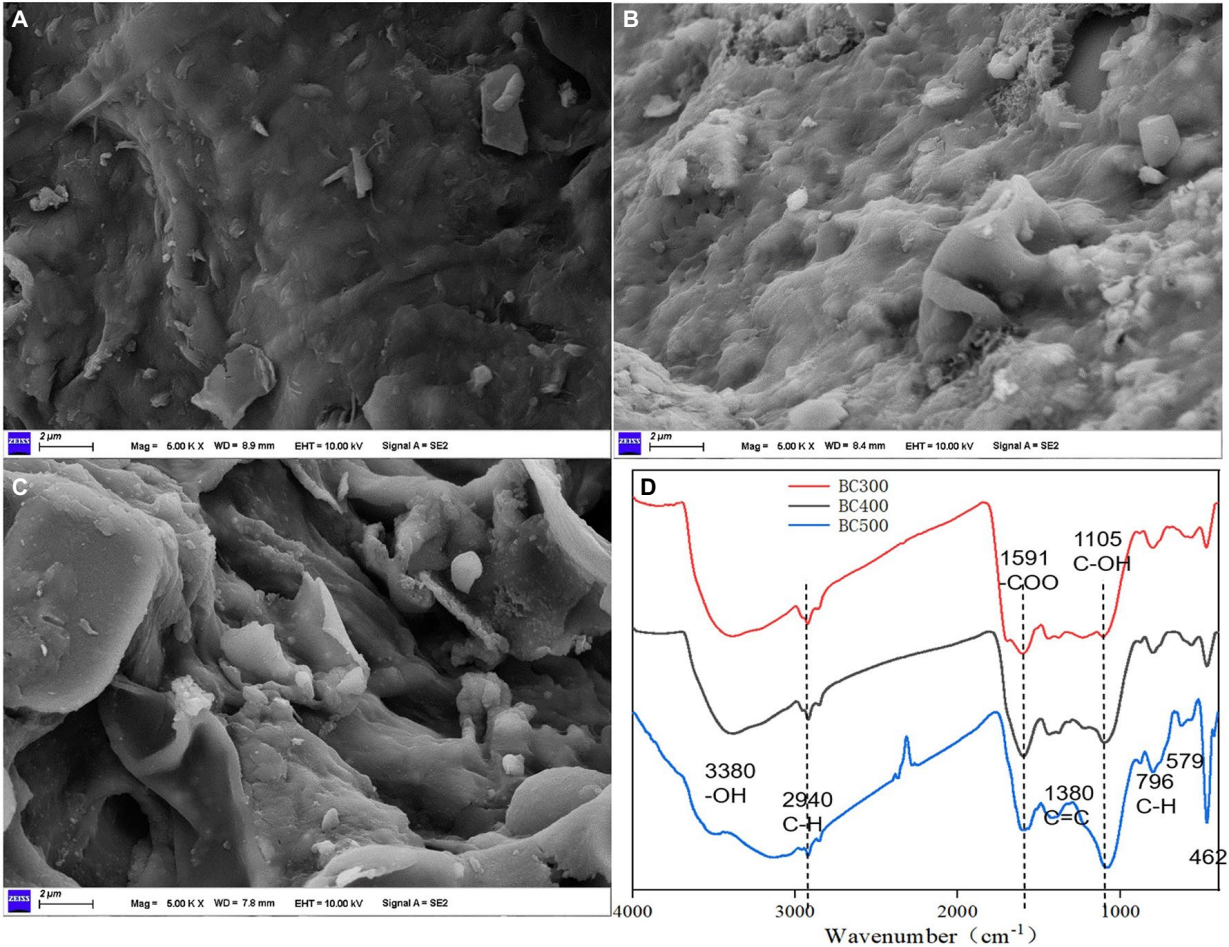
SEM images of BC300 **(A)**, BC400 **(B)**, BC500 **(C)**, and Fourier transform infrared spectroscopy **(D)**. Bar = 2 μm.

SEM images showed the biochar possessed a disintegrated and heterogeneous structure with large macropores (Figure 1). The surface of the BC300 was smooth and flat, and the surface structure of the corn stalk showed reduced damage, owing to insufficient pyrolysis (Figure 1A). The surface of the BC400 was rough, and the stalk surface structure was partially damaged (Figure 1B). However, because of adequate pyrolysis, the surface structure of the BC500 stalk underwent complete destruction, and internal carbonized fiber bundles led to acquiring an intricate surface image (Figure 1C). Predominant macropores on the biochar surface were responsible for the increase in soil water holding capacity and sorption capacity, resulting in the immobilization of toxic metals in extremely polluted soils. Therefore, BC500 could provide increased binding sites for more functional groups, and exhibited a stronger heavy metal ion-adsorption capacity than BC400 and BC300.

FTIR spectra revealed bands at 796, 779, 1,105, and 1,319 cm^−1^, which were allocated to the C–H, Si–O, C–OH, and C=C stretches, respectively (Figure 1D). The presence of another important –COO functional group in aromatic rings was attributed to the 1,591 cm^−1^ wavenumber. The bands at 1,380, 1,435, and 2,360 cm^−1^ indicated the presence of C=C, –CH_2_–, and O=C=O functional groups, respectively. Few common functional groups, such as aliphatic (C–H) and hydroxyl (–OH) stretches, were present at the 2,940, 3,286, and 3,348 cm^−1^ wavenumber, respectively.

### Changes in Foxtail Millet After Biochar Application

#### Growth Characteristics and Biomass

Biochar application enhanced plant growth, including plant height, stem diameter, and stem weight, by 6.74%–11.18%, 29.40%–50.24%, and 35.13%–60.81%, respectively, when compared with the growth of the CK group (Table 3). Furthermore, the highest values for plant height, stem diameter, leaf length, leaf width, root weight, stem weight, leaf weight, and spike weight were observed in the BC500 group, compared with to those in the BC400 and BC300 groups. Stem diameter, leaf width, stem weight, leaf weight, and spike weight were significantly higher in the BC500 group than in the BC300 group. Stem weight and spike weight were significantly higher in the BC500 group than in the BC400 group. No significant differences in traits were observed between BC300 and BC400 groups.

**Table 3:** Growth characteristics and biomass of foxtail millet.

Treatments	Plant height (cm)	Stem diameter (mm)	Leaf length (cm)	Leaf width (cm)	Root weight (g/plant)	Stem weight (g/plant)	Leaf weight (g/plant)	Spike weight (g/plant)
CK	93.27b	6.19c	38.97b	2.56b	2.79b	10.59c	4.03b	8.70b
BC300	99.56a	8.01b	40.35ab	2.67b	2.80ab	14.31b	4.16b	8.79b
BC400	101.35a	8.79ab	44.85a	2.71ab	3.00ab	14.54b	5.71ab	8.81b
BC500	103.70a	9.30a	45.93a	2.79a	3.20a	17.03a	6.33a	9.90a

*Lowercase letters in the same column of each treatment are significantly different at p < 0.05*.

#### Leaf Photosynthesis and Gas Exchange

The photosynthetic rate of foxtail millet was 1.70–1.90 times higher in the BC400 and BC500 groups than in the CK group, whereas the photosynthetic rates were similar in the BC300 and CK groups (Table 4). Stomatal conductance was significantly higher in the BC500 group than in the CK and BC300 groups by 52.97% and 44.69%, respectively. However, intercellular CO_2_ concentrations were lower in the biochar-treated groups relative to that in the CK group (36.87%–62.61%), and no significant differences were observed between the three pyrolysis temperature biochar treatments. Furthermore, no significant effects of biochar application on the transpiration rate were detected.

**Table 4:** Effects on photosynthesis of foxtail millet by biochar application.

Treatments	Photosynthetic rate(μmol CO_2_ m^−2^ s^−1^)	Stomatal conductance (mmol H_2_O m^−2^ s^−1^)	Intercellular CO_2_ concentration (μmol CO_2_ mol^−1^)	Transpiration rate (mmol H_2_O m^−2^ s^−1^)
CK	1.49b	9.61b	331.27a	0.138a
BC300	2.29ab	10.16b	123.87b	0.161a
BC400	2.54a	12.85ab	219.15b	0.166a
BC500	2.82a	14.70a	209.13b	0.172a

*Lowercase letters in the same column of each treatment are significantly different at p < 0.05*.

#### Physiological Characteristics

**Figure 2:**
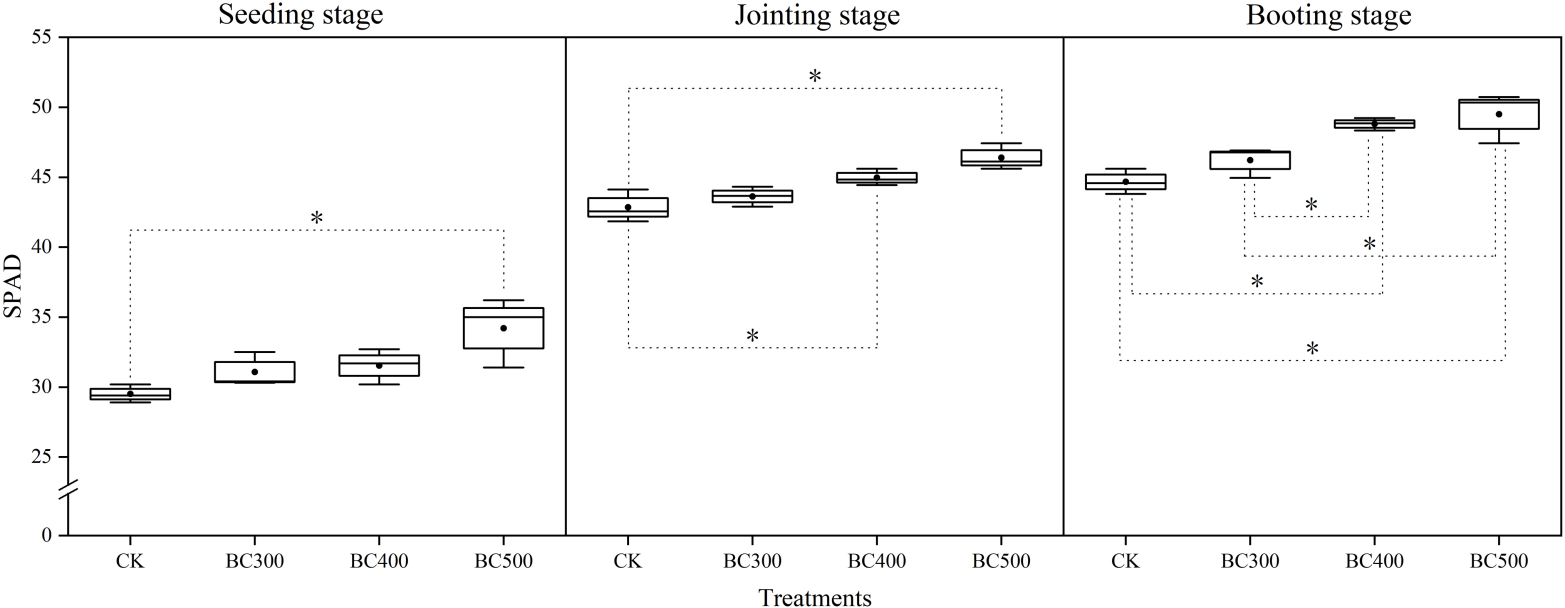
Effects of biochar on SPAD values of leaves. The line and point within the box plot indicate the median and mean values. The boundaries of the box indicate the means ± standard error (SE). The whiskers indicate minimum and maximum. Least significant difference test was adopted with a significance of *p* < 0.05 between treatments shown by *.

Pyrolysis temperature affected SPAD values in foxtail millet following biochar application, and higher SPAD values were observed in the BC400 and BC500 groups than in the CK group at the seedling, jointing, and booting stages, with increases of 10.28%–15.93%, 9.03%–15.10%, and 16.41%–19.14%, respectively (Figure 2). Furthermore, similar SPAD values were observed between the BC300 and CK groups and between the BC400 and BC500 groups. At the seedling and jointing stages, there were no significant differences in SPAD values between the BC300 and BC400 groups; however, the SPAD value was 9.65% higher in the BC400 group than in the BC300 group at the booting stage.

Cd stress was alleviated by biochar application, owing to active oxygen removal by the action of antioxidant enzymes and nonenzymatic systems (Table 5). Lower H_2_O_2_ content and O_2_^−^ production rates were observed in the BC400 and BC500 groups than in the CK group, with decreases of 24.44%–25.41% and 12.20%–21.13%, respectively. Moreover, no significant differences were detected in H_2_O_2_ and O_2_^−^ production rates between the CK and BC300 groups or between the BC400 and BC500 groups. Similarly, 19.76%–43.11% lower MDA content was detected after biochar treatments compared to the CK group, and higher MDA content was detected in the BC300 group than in the BC400 and BC500 groups, with increases of 22.01% and 29.10%, respectively.

**Table 5:** Impact of biochar on the activities of enzymatic and nonenzymatic antioxidants.

Treatments	TP (g kg^−1^)	Pro (μg g^−1^)	MDA (μmol g^−1^)	H_2_O_2_ (mmol g^−1^prot)	O_2_^−^ production rate (U g^−1^prot)	SOD (U mg^−1^prot)	POD (U mg^−1^prot)	CAT (U mg^−1^prot)	GR (U g^−1^prot)
CK	8.27a	170.83a	3.34a	36.99a	292.75a	218.26a	16.89a	2.21a	0.51a
BC300	8.77a	156.70a	2.68b	34.65a	267.13ab	214.13a	16.40ab	1.91ab	0.30b
BC400	8.86a	123.71b	2.09c	27.95b	257.03bc	193.04b	14.15c	1.82b	0.20b
BC500	8.89a	87.75c	1.90c	27.57b	230.89c	177.58b	14.65bc	1.83b	0.21b

*Lowercase letters in the same column of each treatment are significantly different at p < 0.05. TP, total protein; Pro, proline; MDA, malondialdehyde; O_2_^−^, superoxide anion, SOD, superoxide dismutase; POD, Peroxidase; CAT, catalase; GR, glutathione reductase*.

Lower Pro content, and lower SOD, CAT, and POD activities were observed in the BC400 and BC500 groups than in the CK groups, although these indicators showed similar trends in the CK and BC300 groups. No significant differences in SOD, CAT, and POD activities were found between the BC400 and BC500 groups.

Lower Pro content was detected in the BC500 group than in the BC400 group, with a decrease of 29.07%. Lower GR content was observed in biochar treatments than in the CK group, with decreases of 41.78%–60.78%, and no significant differences were observed between biochar treatments. However, no effects of biochar application on TP were observed.

#### Gene Expression

**Figure 3:**
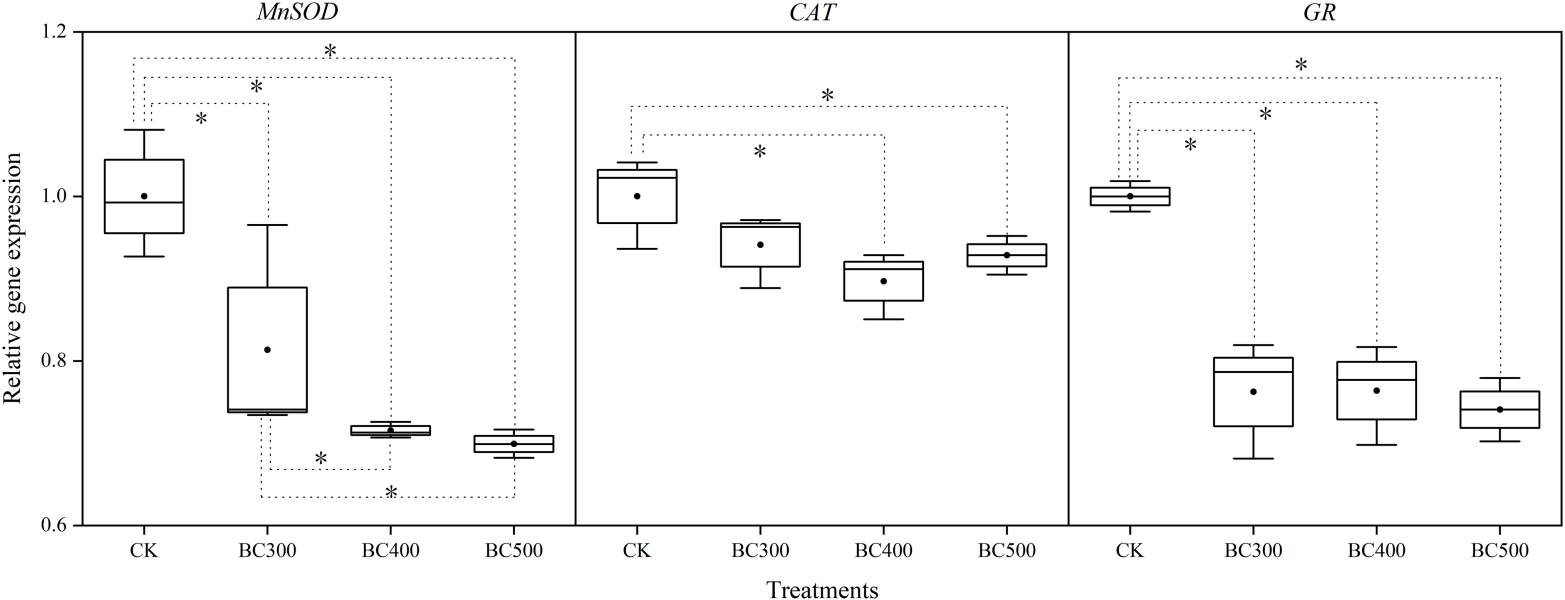
Effects of biochar on *MnSOD*, *CAT*, and *GR* expression. The line and point within the box plot indicate the median and mean values. The boundaries of the box indicate the means ± standard error (SE). The whiskers indicate minimum and maximum. Least significant difference test was adopted with a significance of *p* < 0.05 between treatments shown by *.

Gene expression levels varied, with patterns similar to those of enzyme activity measurements (Figure 3). *MnSOD* and *GR* levels decreased following application of biochar with increased pyrolysis temperatures, compared with those observed in the CK group. Lower *MnSOD* expression was observed in the BC400 and BC500 groups than in the BC300 group, with decreases between 12.06% and 14.04%, respectively. No significant differences were observed between the BC400 and BC500 groups. However, no significant differences in *GR* and *CAT* expression were detected between biochar treatments. Furthermore, lower *CAT* expression was detected in the BC400 and BC500 groups than in the CK group, with decreases between 10.31% and 8.16%, respectively.

#### Cd/Zn Absorption and Distribution

**Figure 4:**
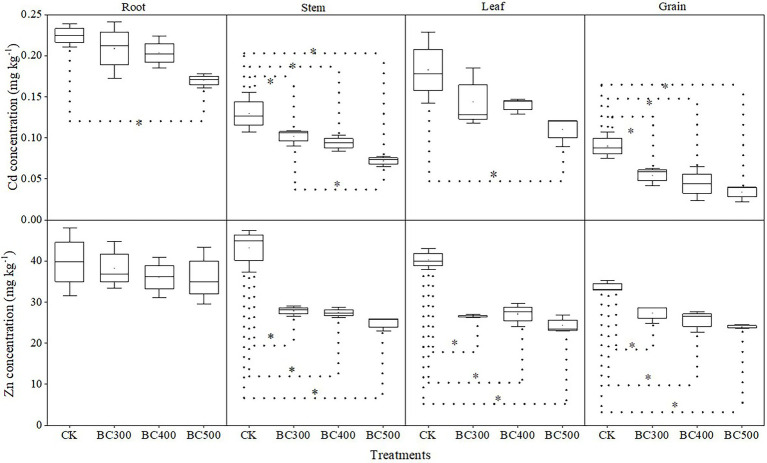
Cd and Zn contents in various organs of foxtail millet. The line and point within the box plot indicate the median and mean values. The boundaries of the box indicate the means ± standard error (SE). The whiskers indicate minimum and maximum. Least significant difference test was adopted with a significance of *p* < 0.05 between treatments shown by *.

The reducing effects of biochar on Cd/Zn content in plants varied with different pyrolysis temperatures and indicated differences in various plant organs (Figure 4). Lower Cd content in the stem (21.43%–44.49%) and grain (39.71%–62.19%) was observed in biochar treatments than in the CK group, and no significant differences in grains were observed among the BC300, BC400, and BC500 groups. Lower Cd content was detected in the stems of the BC500 group compared to those of the BC300 group, with a decrease of 36.91%, and no significant differences were observed between the BC400 and BC500 groups. Furthermore, lower Cd content in the root and leaf was detected in the BC500 group than in the CK group, with decreases of 22.73% and 38.89%, respectively, and no significant differences were observed among biochar treatments.

Zn content in the stem, leaf, and grain was also reduced by biochar application, and the decreases were not dependent on pyrolysis temperature. However, no significant effects on Zn content were detected in the root following biochar application. Notably, the Cd and Zn content in the grains was 39.71%–62.29% and 19.11%–29.00% lower, respectively, than those in the CK group.

#### Bioconcentration and Translocation Factors of Cd/Zn

Biochar application decreased the BF of Cd and Zn and the TF of Cd in the shoots (Table 6). The BF and EC of Cd in roots from the BC500 group were lower than those in the CK group, with decreases of 25.93% and 19.05%, respectively, and no significant differences between the BC300, BC400, and CK group were observed. Notably, in the roots, no significant differences in BF, TF, and EC were observed between the three biochar treatments, whereas BF in the shoots in the BC500 group was lower than that in the shoots of the BC300 group by 27.27%.

**Table 6:** Bioconcentration factors (BFs), translocation factors (TFs), and extraction coefficients (ECs) of foxtail millet.

	Treatments	Bioconcentration factor		Translocation factor	Extraction coefficient (%)
		Root	Shoot		
Cd	CK	0.27a	0.15a	0.55a	0.84a
	BC300	0.25ab	0.11b	0.45b	0.75ab
	BC400	0.25ab	0.11bc	0.43b	0.76ab
	BC500	0.20b	0.08c	0.40b	0.68b
Zn	CK	0.10a	0.10a	1.02a	0.50a
	BC300	0.09a	0.07b	0.73ab	0.42a
	BC400	0.09a	0.07b	0.75ab	0.44a
	BC500	0.09a	0.06b	0.70b	0.46a

*Lowercase letters in the same column of each treatment are significantly different at p < 0.05*.

The BF of Zn was significantly lower than Cd, particularly in the roots. However, the TF of Zn was significantly higher than Cd. Similar BF and EC values of Zn were detected in the roots across all biochar treatments compared with those in the CK group. The shoots had lower BF values for Zn in all three biochar treatments compared with those observed in the CK group, with decreases of 30.00%–40.00%, and no significant differences were detected between the three biochar treatments. Moreover, the TF value in the BC500 group was lower than that in the CK group, and no significant differences were observed between the three biochar treatments.

### Soil Characteristics

#### Total and Available Cd/Zn Content

**Figure 5:**
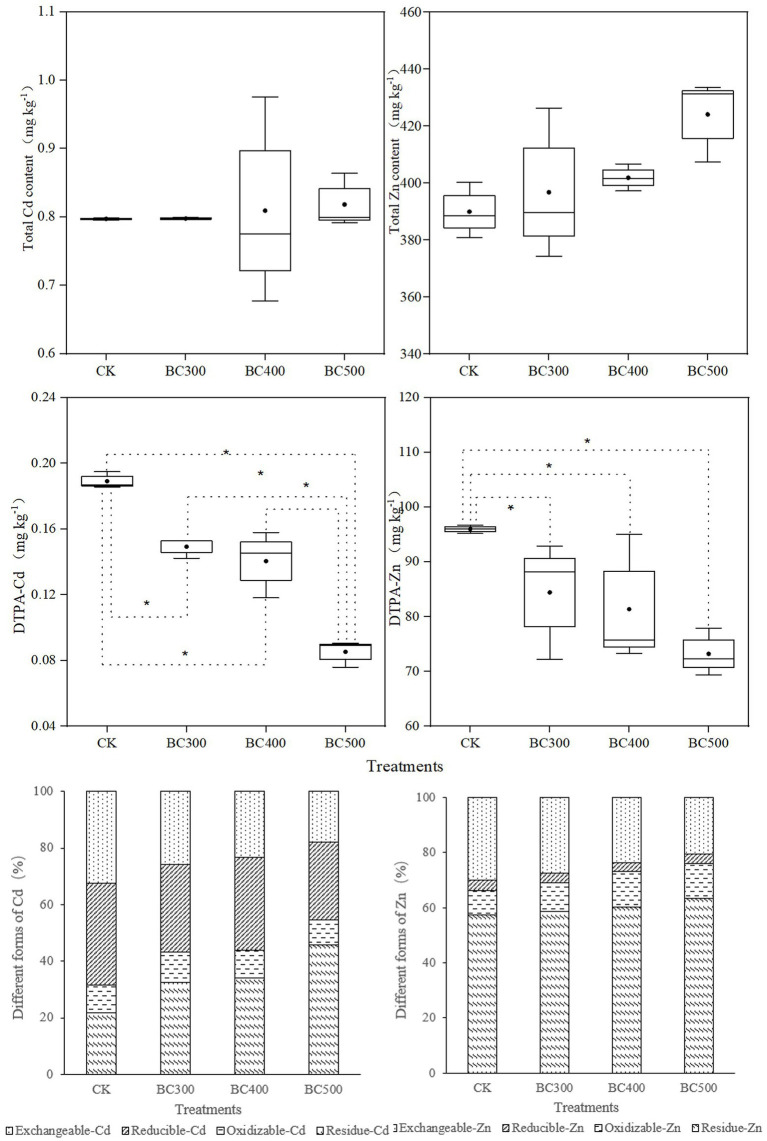
Effects of biochar on the contents of total Cd, total Zn, DTPA-Cd, and DTPA-Zn and the proportions of different forms of Cd and Zn. The line and point within the box plot indicate the median and mean values. The boundaries of the box indicate the means ± standard error (SE). The whiskers indicate minimum and maximum. Least significant difference test was adopted with a significance of *p* < 0.05 between treatments shown by *.

No significant effects of biochar application on total Cd and Zn content were detected (Figure 5). However, 21.05%–52.63% lower DTPA-Cd and 12.03%–23.70% lower DTPA-Zn content was detected in all biochar treatments compared with those observed in the CK group, and the decrease in DTPA-Cd in the BC500 group was greater than that in the BC300 (21.42%) and BC400 groups (26.05%). Furthermore, no significant differences in DTPA-Zn content were detected between the three biochar treatments, and no differences in DTPA-Cd were detected between the BC300 and BC400 groups.

In the CK treatment, most of the Cd content existed in the form of exchangeable-Cd (32.51%) and reducible-Cd (35.85%). The content of exchangeable-Cd and reducible-Cd decreased upon biochar application, whereas the content of residue-Cd significantly increased. The highest proportion of residue-Cd (44.66%) and the lowest proportion of exchangeable-Cd (17.85%) and reducible-Cd (27.59%) were detected in the BC500 group. Furthermore, the lowest proportion of oxidizable-Cd was also observed in the BC500 group.

Exchangeable-Zn content was reduced, and the content of oxidizable-Zn and residue-Zn increased upon biochar application. The highest proportion of residue-Zn and the lowest proportion of exchangeable-Zn were also observed in the BC500 group. Most Zn content existed in the residue form, accounting for 57.33%–63.31% of the total soil zinc content. The second most abundant form was exchangeable-Zn, accounting for 20.56%–30.01% of total zinc content. Reducible-Zn and oxidizable-Zn accounted for only a small proportion of the zinc content, i.e., 3.16%–3.71% and 8.95%–12.92%, respectively.

#### Soil pH, CEC, and Nutrients

**Figure 6:**
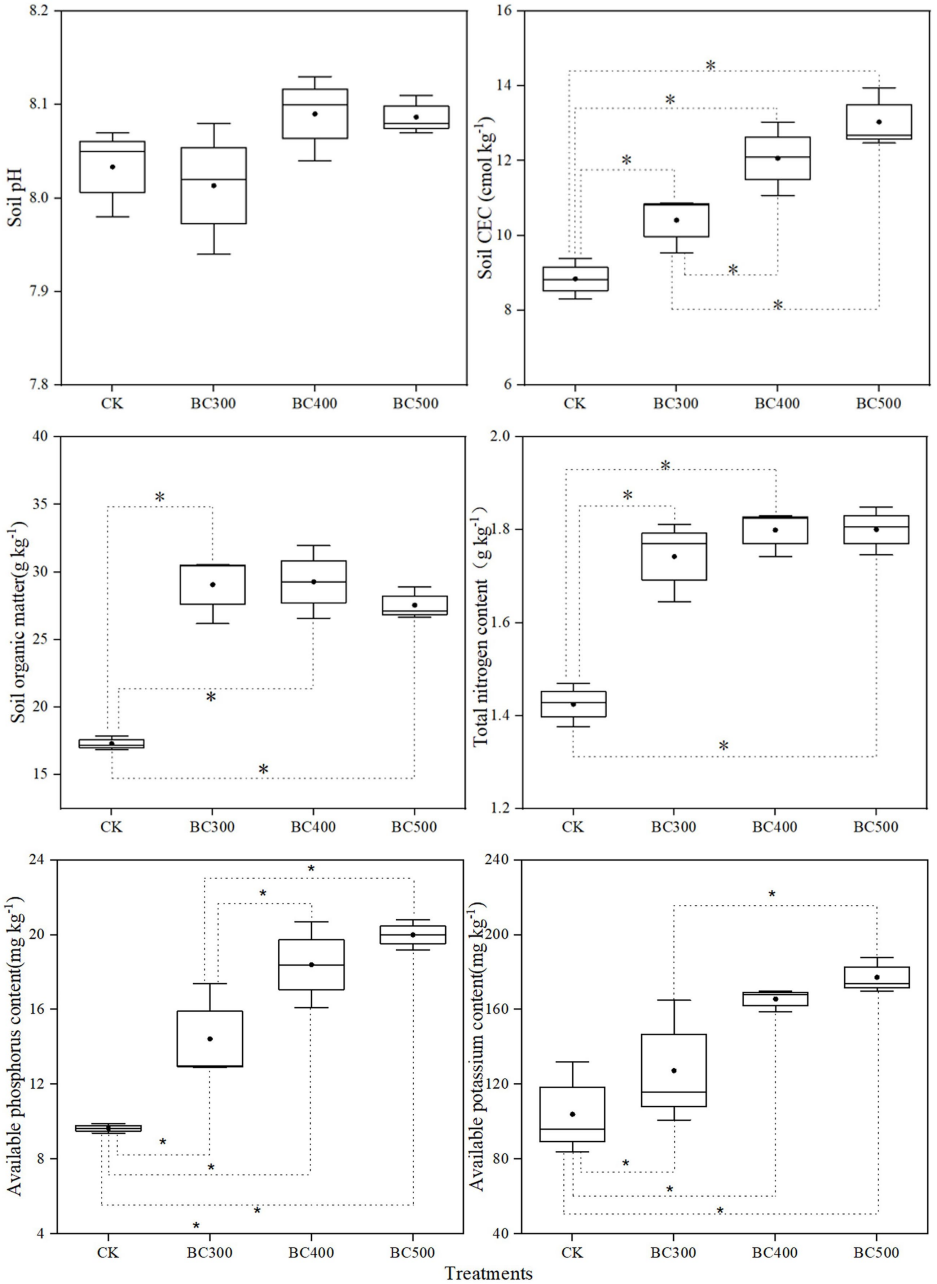
Effects of biochar on soil pH, CEC, and organic matter and nutrient contents in post-harvest soil. The line and point within the box plot indicate the median and mean values. The boundaries of the box indicate the means ± standard error (SE). The whiskers indicate minimum and maximum. Least significant difference test was adopted with a significance of *p* < 0.05 between treatments shown by *.

No significant differences in pH values were detected between the CK and biochar treatment groups (Figure 6). Higher CEC, organic matter, total nitrogen, and available P were observed in the biochar treatment groups than in the CK group. Lower CEC and available P were detected in the BC300 group than in the BC400 and BC500 groups. Furthermore, no significant differences were observed in soil organic matter or total nitrogen content between biochar treatments. However, available K was higher in the BC400 and BC500 groups than in the CK group, with increases of 59.30% and 70.51%, respectively. No significant differences were observed between the BC400 and BC500 groups.

#### Soil Enzymatic Activity

**Figure 7:**
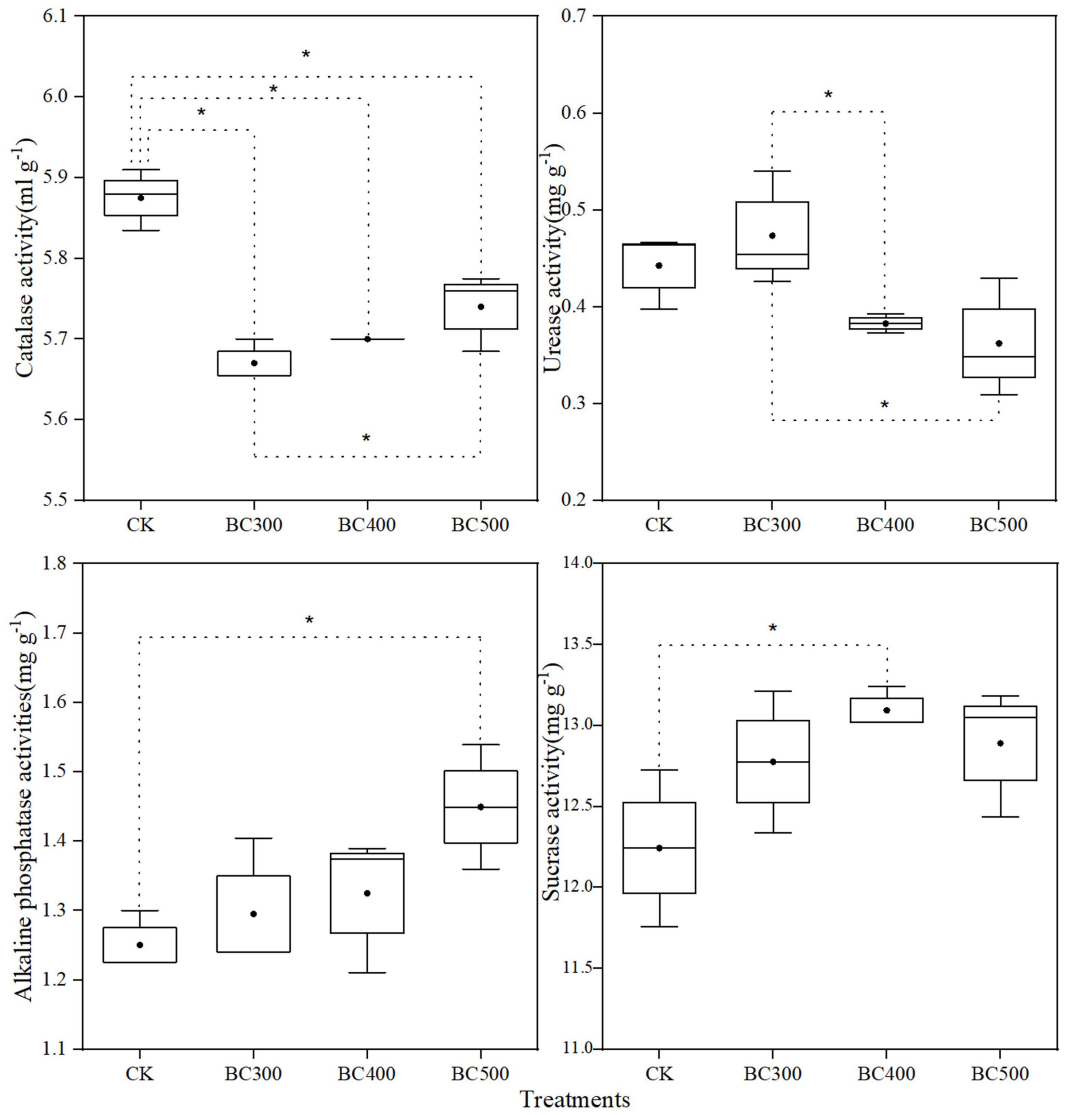
Effects of biochar on soil catalase, urease, alkaline phosphatase, and sucrase activities. The line and point within the box plot indicate the median and mean values. The boundaries of the box indicate the means ± standard error (SE). The whiskers indicate minimum and maximum. Least significant difference test was adopted with a significance of *p* < 0.05 between treatments shown by *.

CAT activity following biochar treatment was reduced by 2.38%–3.57% compared to that observed in the CK group (Figure 7). CAT activity in the BC300 group was 1.22% lower than that in the BC500 group. Compared with the BC300 group, urease activity was significantly reduced in the BC400 and BC500 groups, with decreases of 19.15% and 23.40%, respectively. High alkaline phosphatase activity was observed in the BC500 group, and sucrase activity was enhanced in the BC400 group. Differences in alkaline phosphatase and sucrase activities between biochar treatments were not significant.

### Diversity and Composition of Microbial Communities in Soil

The soil bacterial and fungal communities showed different profiles with the addition of biochar (Table 7). Compared to the CK treatment, the BC500 treatment significantly increased the number of bacterial OTUs. BC300 and BC500 treatments significantly increased the number of fungal OTUs. There were no significant differences between biochar treatments in terms of bacterial and fungal OTUs. The Chaol index of the soil bacterial communities and Chaol and Simpson indices of the soil fungal communities were significantly higher in biochar-treated samples than in the CK group. Furthermore, a higher Shannon index was detected in the BC400 and BC500 groups compared to the CK group, with increases of 20.90% and 23.77%, respectively. However, no significant differences were observed in the Simpson and Shannon indices for soil bacterial communities between the three treatments.

**Table 7:** Impact of biochar on the operational taxonomic unit (OUT) and α diversity indices.

Treatments	Bacterial community				Fungal community			
	OTUs	Chao1	Simpson	Shannon	OTUs	Chao1	Simpson	Shannon
CK	5692.33b	6018.98b	1.00a	10.81a	282.67b	282.78c	0.91c	4.88b
BC300	5945.33ab	6778.07a	1.00a	10.96a	368.00a	368.50b	0.94b	5.30ab
BC400	6123.33ab	6952.86a	1.00a	10.96a	307.67ab	374.88b	0.97a	5.90a
BC500	6113.67a	6787.24a	1.00a	11.01a	386.33a	410.25a	0.96ab	6.04a

*Lowercase letters in the same column of each treatment are significantly different at p < 0.05*.

**Figure 8:**
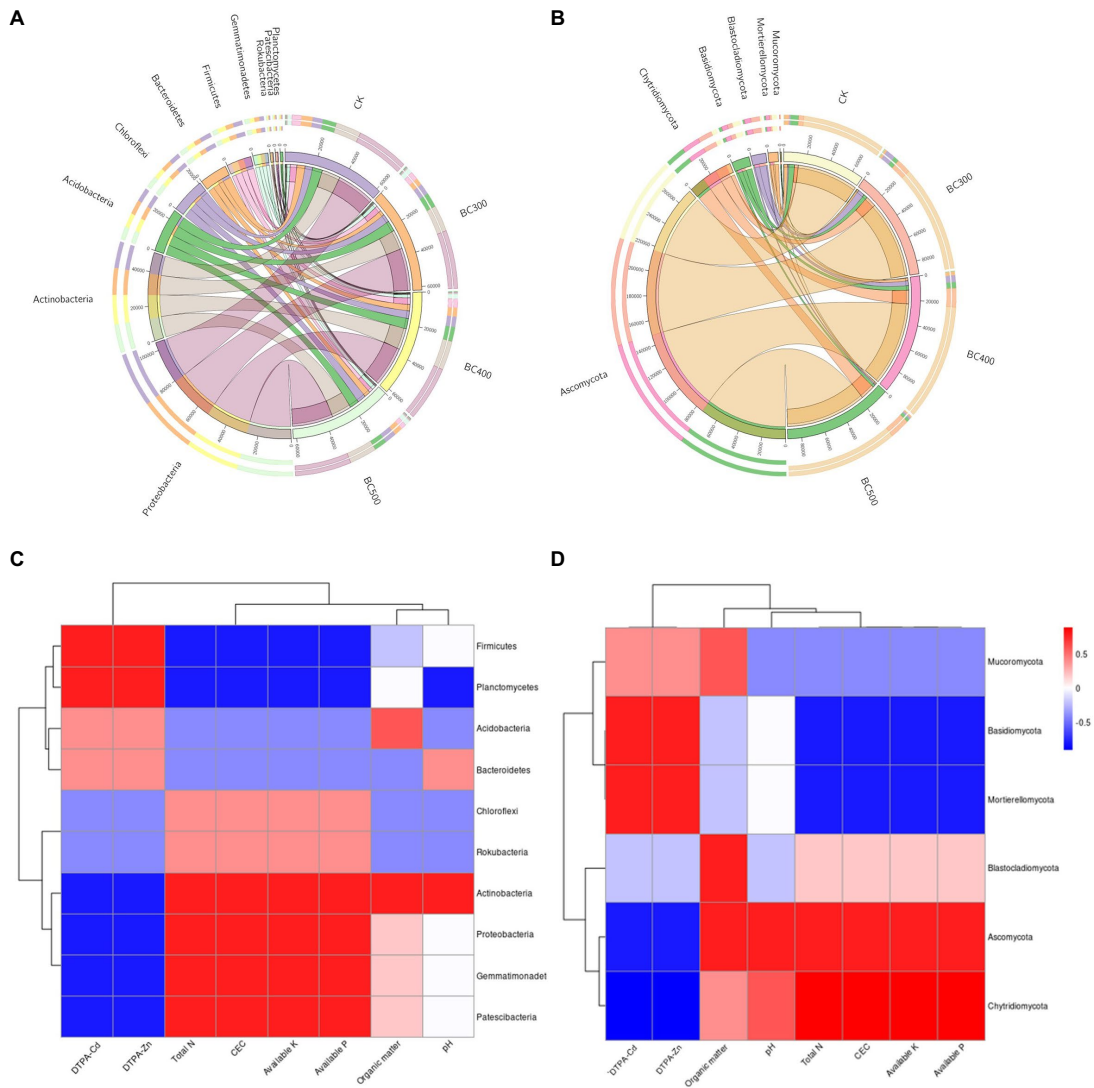
The compositions of bacterial **(A)** and fungal **(B)** communities at the phylum level. Illustration of the Spearman correlation heatmap between soil environmental factors and the predominant bacterial phyla **(C)** and fungal phyla **(D)**.

In this study, the dominant soil bacterial phyla were Proteobacteria (39.58%–42.36% relative abundance), Actinobacteria (19.18%–21.19%), Acidobacteria (9.50%–10.48%), Chloroflexi (7.56%–8.84%), Bacteroidetes (5.15%–8.43%), Firmicutes (4.10%–7.07%), Gemmatimonadetes (3.32%–3.88%), Rokubacteria (0.69%–0.77%), Patescibacteria (0.58%–0.77%), Planctomycetes (0.56%–0.77%), Nitrospirae (0.48%–0.68%), and Cyanobacteria (0.32%–0.59%; Figure 8A). The dominant soil fungal phyla were Ascomycota (76.41%–80.72%), Basidiomycota (2.38%–7.65%), Blastocladiomycota (1.25%–6.83%), Chytridiomycota (4.67%–14.45%), Mortierellomycota (1.18%–4.87%), and Mucoromycota (0.02%–0.38%; Figure 8B). Biochar application exerted significant effects on the bacterial taxa distribution. BC300 treatment significantly decreased the relative abundances of Firmicutes and Bacteroidetes, and increased the relative abundance of Patescibacteria. Lower relative abundances of Firmicutes, Planctomycetes, and Nitrospirae, and higher relative abundance of Patescibacteria and Cyanobacteria were detected in BC500 treatments. Regarding the fungal taxa distribution, the effects of biochar treatment were more dramatic. All biochar treatments significantly enhanced the relative abundance of Chytridiomycota and reduced the relative abundances of Basidiomycota and Mortierellomycota. A high relative abundance of Blastocladiomycota was observed in the BC300 and BC400 groups. The relative abundance of Mucoromycota was increased in the BC300 group, whereas that in the BC500 group was significantly reduced.

The relationships between soil environmental factors and the preponderant bacterial phyla (top 10) and fungal phyla (top 6) are shown in Figures 8C,D. Four bacterial phyla and two fungal phyla in the soil showed positive correlations with both DTPA-Cd and DTPA-Zn, but negative correlations with total N, CEC, available P, and available K. Six soil bacterial phyla and three soil fungal phyla showed positive correlations with soil total N, CEC, available P, and available K, but showed negative correlations with DTPA-Cd and DTPA-Zn. The bacterial phyla Acidobacteria and Actinobacteria, and fungal phyla Ascomycota and Blastocladiomycota, showed positive correlations with soil organic matter. Acidobacteria and Ascomycota showed positive correlations with soil pH, whereas Planctomycetes showed a negative correlation.

## Discussion

In this study, biochar showed effective passivation effects on heavy metals by transforming the exchangeable form with high biotoxicity to the inert form with weak biotoxicity. Adsorption, coprecipitation and complex-formation maybe the main heavy metal passivation mechanisms of biochar due to its large surface area, as well as high ion exchange capacity and abundant functional groups. Compared with BC300 and BC400 treatments, BC500 showed better passivation effects on soil Cd and Zn, which was consistent with the findings reported by Chen H. H. et al. (2020). Thus, biochar played an active role in promoting heavy metal transformation of exchangeable components to their stable forms.

Additionally, we showed that the passivation ratio of Cd was significantly higher than Zn. Therefore, biochar application may be a valid approach for reducing heavy metal toxicity. Lower Cd and Zn concentrations were recorded in biochar-treated groups than in the CK group across all foxtail millet organs. These observations were consistent with findings reported in rice (Muhammad et al., 2018), where biochar application significantly decreased the Cd concentrations in the roots and shoots. Houben et al. (2013) also demonstrated that biochar application could reduce Cd and Zn concentrations in plants. In the CK group, the average Cd concentration in the grains was similar to the standard Cd content, which is limited to 0.1 mg/kg (GB2762-2017). Biochar is an effective ameliorant; its application reduces the risk of excessive Cd accumulation in food grown on Cd-contaminated soils. Across all three biochar applications, BF of Cd and Zn in the shoots were significantly reduced compared to the roots, and reduced the TF of Cd and Zn across the entire foxtail millet, compared to CK. This indicated that biochar application led to the fixation of Cd and Zn in the roots and prevented further absorption and transport of heavy metals in plants. Moreover, the TF of Zn was significantly higher than Cd. This suggests that Zn possesses a stronger migration ability in plants than Cd.

Cd can reduce the growth and biomass of plants in several food and cash crops, such as wheat (Abbas et al., 2017), maize (Akhtar et al., 2017), and rice (Liu et al., 2009). In plants, Cd binds the essential elements in metalloproteins and the sulfhydryl groups in proteins or enzyme active sites, further inhibiting plant growth (Yang et al., 2011). In the present study, growth indicators and biomass characteristics were significantly enhanced by biochar application compared with those in the CK group. Our results were similar to those reported in peanuts, in which biochar application also enhanced the biomass and physiological quality (Chen X. et al., 2020). This result can be attributed to the inactivation ability of biochar exerted on Cd and Zn. Among the three biochar treatments, the highest plant height, stem diameter, leaf length, leaf width, and biomass were recorded in the BC500 group. Therefore, this phenomenon presented the excellent passivation effects of BC500 relative to BC300 and 400.

Shimshi and Ephrat (1975) demonstrated positive correlations among grain yield, photosynthetic rate, and stomatal conductance. Photosynthetic processes are considered vital indicators for assessment of the inhibitory effects of biological or abiotic stress on plants (Roy et al., 2022a). Chlorophyll content also plays an important role in photosynthesis in plants and has therefore been used to assess the health status of plants (Zeng et al., 2019). Cd toxicity contributes to the impairment of chlorophyll pigments, photosynthesis, transpiration, stomatal conductance, and other physiological and photosynthetic processes, by inflicting damage to the thylakoid membrane and chlorophyll precursor formation (Vaculík et al., 2015). Li et al. (2021) reported that chlorophyll content reflects the level of photosynthesis reduction occurring due to Cd stress. Tian et al. (2016) suggested that NaHS alleviates Cd-induced damage by strengthening photosynthesis. In this study, chlorophyll content, photosynthesis, and gas exchange rates were improved across all biochar treatments, particularly in the BC500 group, owing to Cd immobilization. These findings were consistent with the observations reported by Muhammad et al. (2019), who showed that adding biochar in Cd-contaminated soils increased the photosynthetic rate, transpiration rate, and stomatal conductance of wheat.

Under adverse conditions of environmental stress, plants can develop various mechanisms to alleviate physiological injury (Roy et al., 2021b, 2022b). Existing literature has indicated that the content of MDA and H_2_O_2_ and the production rate of O_2_^−^ are indicators of plant cell damage, and antioxidant enzymes, such as SOD, POD, CAT, and GR, facilitate the elimination of reactive oxygen species (ROS) and confer protection to cells against stress-induced damage (Shen et al., 2020). In the current study, the content of Pro, MDA, and H_2_O_2_ decreased following biochar treatment, and the lowest content levels of Pro, MDA, and H_2_O_2_ were observed in the BC500 group. The activities of SOD, POD, CAT, and GR were also significantly reduced, among which the reduction in GR was particularly dramatic. GR, a key enzyme in the ascorbate-glutathione cycle, catalyzes the conversion of oxidized glutathione to reduced glutathione and plays crucial roles in detoxifying Cd-induced ROS to alleviate stress (Zhan et al., 2017). The activity of antioxidant enzymes showed positive correlations with MDA and H_2_O_2_ content. The decline in antioxidant enzyme activity indicated that biochar alleviated the biological toxicity of Cd by causing its inactivation in the soil. Furthermore, in our study, the expression levels of *MnSOD*, *CAT*, and *GR* were downregulated by biochar application, owing to the reduced phytotoxicity of Cd and the levels of ROS in plants.

Soil microorganisms may play important roles in the immobilization and precipitation of heavy metals in the soil. Hence, changes in the diversity and composition of microbial communities in soil using biochar application have attracted considerable attention (Gul et al., 2015). In the current study, biochar application significantly increased the abundance of soil bacteria and fungi, and the species richness of fungi; however, the effects on the species richness of bacteria were not significant. These observations follow the findings reported by Yao et al. (2017). With the addition of biochar, two additional fungal phyla (Glomeromycota and Rozellomycota) were observed, compared with those in control soil. Application of biochar increased the presence of specific taxa, which contributed to the richness of soil fungal communities. Biochar affects the relative abundance of dominant soil bacterial phyla. Consistent with the observations reported by Wang et al. (2021), Proteobacteria showed the highest abundance of bacterial phyla in our study. Their unique metabolic and ecological ability to adapt to the extreme environments of heavy metal-contaminated soil and reduce the toxicity of heavy metals highlighted these organisms as the most metal-tolerant bacterial phylum present in heavy metal-contaminated sites (Guo et al., 2019). The effects of biochar on the relative abundance of dominant soil fungal phyla were more significant. The relative abundances of the five fungal phyla varied significantly, indicating that the soil fungi showed major responses to biochar. Although biochar has a good passivation effect on Cd and Zn, the passivation effects in other soil types were not clear. Furthermore, modified biochar with good passivation effect needs to be further explored.

In this study, biochar was shown as an effective passivation material for soil Cd and Zn combined pollution, and the alleviation damage on heavy metal stress depended on the pyrolysis temperature. Furthermore, the passivation ratio of Cd was significantly higher than Zn. The DTPA-Cd and DTPA-Zn content levels were significantly reduced upon biochar treatment, owing to the transformation of exchangeable components to stable forms. Adding biochar also promoted the growth of foxtail millet, alleviated oxidative stress, and reduced the bioaccumulation of Cd and Zn. Moreover, biochar application significantly increased the abundance of soil bacterial and fungal phyla and enhanced the species richness of fungi. Furthermore, the passivation effect of biochar exerted on heavy metals in the soil was affected by the pyrolysis temperature of biochar treatment. Of the three pyrolysis temperature-based biochar treatments conducted in this experiment, elevated pyrolytic temperatures increase the activation of functional groups on the biochar surface, which increases its ability to form complexes with heavy metals. Therefore, BC500 is an effective tool to remediate Zn and Cd contamination in foxtail millet grown in arid and alkaline regions.

## Data Availability Statement

The original contributions presented in the study are publicly available. This data can be found at: National Center for Biotechnology Information (NCBI) BioProject database under accession numbers PRJNA815490 and PRJNA815492.

## Author Contributions

YL and YZ: conceptualization, funding acquisition, and writing–review and editing. XK and NG: data curation and investigation. XL and JY: formal analysis. QY: resources. HW: software. HP: validation. XK: writing–original draft. All authors contributed to the article and approved the submitted version.

## Funding

This work was supported by the Modern Agriculture Industrial Technology Systems Project of Shandong Province (SDAIT-15-04), and Major Basic Research Projects of Shandong Natural Science Foundation (ZR2018ZC2363). The sponsor(s) had no role in the study design; collection, analysis, interpretation of data; writing of the report, and in the decision to submit the article for publication.

## Conflict of Interest

The authors declare that the research was conducted in the absence of any commercial or financial relationships that could be construed as a potential conflict of interest.

## Publisher’s Note

All claims expressed in this article are solely those of the authors and do not necessarily represent those of their affiliated organizations, or those of the publisher, the editors and the reviewers. Any product that may be evaluated in this article, or claim that may be made by its manufacturer, is not guaranteed or endorsed by the publisher.
